# Dose-response efficacy of horticultural therapy for geriatric depression: a systematic review and meta-analysis of randomized controlled trials

**DOI:** 10.3389/fpubh.2026.1824111

**Published:** 2026-07-17

**Authors:** Qing Li, Yujie Wang, Xia Wang, Xuna Han, Naiwen Hu

**Affiliations:** 1 Jinan Development Service Center of Parks, Jinan, China; 2 Department of Rheumatology and Immunology, Shandong Provincial Hospital Affiliated to Shandong First Medical University, Jinan, China

**Keywords:** dose–response, geriatric depression, horticultural therapy, meta-analysis, restricted cubic splines

## Abstract

**Background:**

Horticultural therapy (HT) is a promising non-pharmacological intervention for geriatric depression. However, the optimal “dose” required for maximum clinical efficacy remains poorly defined.

**Objective:**

To evaluate the efficacy of HT in alleviating geriatric depression and to quantify the dose–response relationship between intervention intensity and clinical outcomes.

**Methods:**

A systematic search was conducted across eight databases (including PubMed, Embase, and Cochrane Library, etc) for randomized controlled trials (RCTs) published up to 24 February 2026. Standardized mean differences (SMD) were pooled using a random-effects model. The dose–response relationship was modeled using restricted cubic splines (RCS).

**Results:**

A total of eight RCTs involving 585 participants were initially identified. Systematic sensitivity analysis identified one study as a significant statistical outlier. After its exclusion, the final meta-analysis included seven studies with 435 participants. HT was associated with a significant reduction in geriatric depression scores (SMD = −0.52; 95% CI: −0.86 to −0.18; *p* = 0.0078). Heterogeneity remained moderate (I^2^ = 65.6%). The RCS model revealed an exploratory, hypothesis-generating non-linear dose–response relationship (*p* = 0.076), suggesting that the antidepressant benefit might peak at a cumulative dose of approximately 700–800 min. However, this finding should be interpreted with caution due to the limited number of studies. Subgroup analysis indicated that the GDS-30 scale showed a marginally significant trend toward higher sensitivity to treatment effects than the GDS-15 (*p* = 0.055). No significant publication bias was detected via Egger’s test (*p* = 0.936) and Begg’s test (*p* = 0.652).

**Conclusion:**

HT is an effective intervention for geriatric depression. A cumulative dose of 700–800 min represents a potential exploratory therapeutic window for maximizing efficacy while minimizing potential fatigue in frail older adults.

**Systematic review registration:**

PROSPERO, CRD420261323722.

## 1 Background

Geriatric depression has emerged as a major global public health challenge, affecting approximately 15% of the older population worldwide (1). It is not merely a transient emotional state, but a debilitating condition associated with significant functional decline, increased risk of chronic physical comorbidities, and elevated mortality rates (2). While pharmacological treatments remain the standard of care, their efficacy in older adults is often limited by polypharmacy, adverse drug reactions, and poor treatment adherence. Consequently, there is an urgent need for robust, non-pharmacological interventions that are both safe and effective for this vulnerable population.

Horticultural therapy (HT), a holistic intervention involving plant-based activities guided by trained professionals, has gained increasing attention as a promising alternative. Unlike conventional physical exercises, HT is a multi-dimensional intervention that integrates physical activity, cognitive stimulation, and social interaction within a natural environment (3). According to the Biophilia Hypothesis and Stress Reduction Theory, the sensory engagement—visual, olfactory, and tactile—inherent in gardening can lower physiological arousal and promote emotional recovery (4, 5). Recent studies have suggested that HT can significantly alleviate depressive symptoms across various geriatric settings, from community-dwelling individuals to those in residential care facilities (3, 6).

However, a critical gap remains in the current literature: the dose–response relationship of horticultural therapy. While the general benefits of HT are well-documented, existing guidelines provide vague recommendations regarding the optimal “dose”—defined by the frequency and cumulative duration of intervention—required to achieve maximum clinical efficacy. Most previous meta-analyses have treated HT as a categorical “yes/no” intervention, failing to account for how different intensities of exposure influence outcomes. Furthermore, for older adults with functional limitations, excessive activity may lead to physical fatigue or diminishing returns, suggesting a potential non-linear relationship between dose and effect (7, 8).

To address these limitations, this study presents a systematic review and the first dose–response meta-analysis using restricted cubic splines (RCS) to quantify the relationship between HT dosage and depression reduction in older adults. By identifying the “sweet spot” of intervention duration and managing study heterogeneity through rigorous outlier detection, we aim to provide a precise “time prescription” for clinical practitioners and older population care providers, thereby maximizing the therapeutic potential of horticultural therapy in geriatric mental health.

## 2 Methods

### 2.1 Registration and guidelines

This systematic review and meta-analysis were conducted in accordance with the PRISMA 2020 (Preferred Reporting Items for Systematic Reviews and Meta-Analyses) statement. The protocol was pre-registered in the PROSPERO database (registration no.: CRD420261323722).

### 2.2 Search strategy

A comprehensive systematic search was performed across eight electronic databases: PubMed, Embase, Web of Science, Cochrane Library, PsycINFO, CNKI, WanFang, and VIP. The search period spanned from database inception to 24 February 2026.

We utilized a combination of MeSH terms and keywords to identify relevant studies. The search strings were constructed using three primary components:

Intervention: “horticultural therapy,” “gardening,” “therapeutic horticulture.”
Condition/Population: “depression,” “depressive symptoms,” “aged,” “elderly,” “older adults.”
Study Design: “randomized controlled trial,” “RCT.”

No language restrictions were applied to minimize publication bias. Manual searches of the reference lists of included studies and relevant reviews were also conducted to ensure a thorough search.

### 2.3 Inclusion and exclusion criteria

Studies were included if they met the following PICOS criteria:

Participants: Older adults (aged ≥ 60 years).
Intervention: Horticultural therapy (HT), including active gardening or passive sensory stimulation with plants.
Comparison: Control groups receiving routine care, no intervention, or waiting-list control.
Outcomes: Depression severity measured by validated scales such as the Geriatric Depression Scale (GDS-15/30).
Study Design: Randomized controlled trials (RCTs).

### 2.4 Data extraction and quality assessment

Two reviewers independently extracted data using a standardized form, including study location, setting, participant characteristics, intervention dose (frequency, duration, and total minutes), and pre−/post-intervention mean and standard deviation (SD).

The risk of bias for each study was assessed using the Cochrane Risk of Bias 2.0 (RoB 2.0) tool, covering domains such as the randomization process, deviations from intended interventions, missing outcome data, measurement of the outcome, and selection of the reported result.

### 2.5 Statistical analysis

All statistical analyses were conducted using R software (version 4.3.1). Given the nature of the included randomized controlled trials (RCTs), the standardized mean difference (SMD) with 95% confidence intervals (CIs) was employed as the primary effect size to account for the different versions of the Geriatric Depression Scale (GDS-15 and GDS-30) used across studies.

#### 2.5.1 Pooling and heterogeneity

A random-effects model (restricted maximum likelihood method) was used for all meta-analyses to accommodate potential clinical and methodological diversity. Heterogeneity was quantitatively assessed using the statistic and Cochrane’s test.

To ensure the robustness of the pooled estimates, outliers were identified and handled based on objective statistical criteria: (1) a standardized residual greater than ±3, and (2) a substantial reduction in the I^2^ statistic (>30%) upon removal during leave-one-out sensitivity analysis (9, 10). This procedure was applied to all preliminary syntheses to address extreme statistical inconsistency.

#### 2.5.2 Dose–response analysis

To explore the non-linear relationship between the total intervention dose and depression reduction, a dose–response meta-analysis was performed using restricted cubic splines (RCS) with three knots. Three knots were selected as they represent the most parsimonious and robust baseline approach for modeling non-linear trends in small sample sizes, effectively avoiding overfitting while adequately capturing potential U-shaped or threshold effects. Non-linearity was formally evaluated using the Wald test. This advanced modeling allows for the identification of potential therapeutic thresholds that linear models might overlook.

#### 2.5.3 Bias assessment

Small-study effects and potential publication bias were evaluated through a combination of visual inspection of funnel plots and statistical confirmation via Egger’s linear regression test and Begg’s rank correlation test.

## 3 Results

### 3.1 Study selection

**Figure 1:**
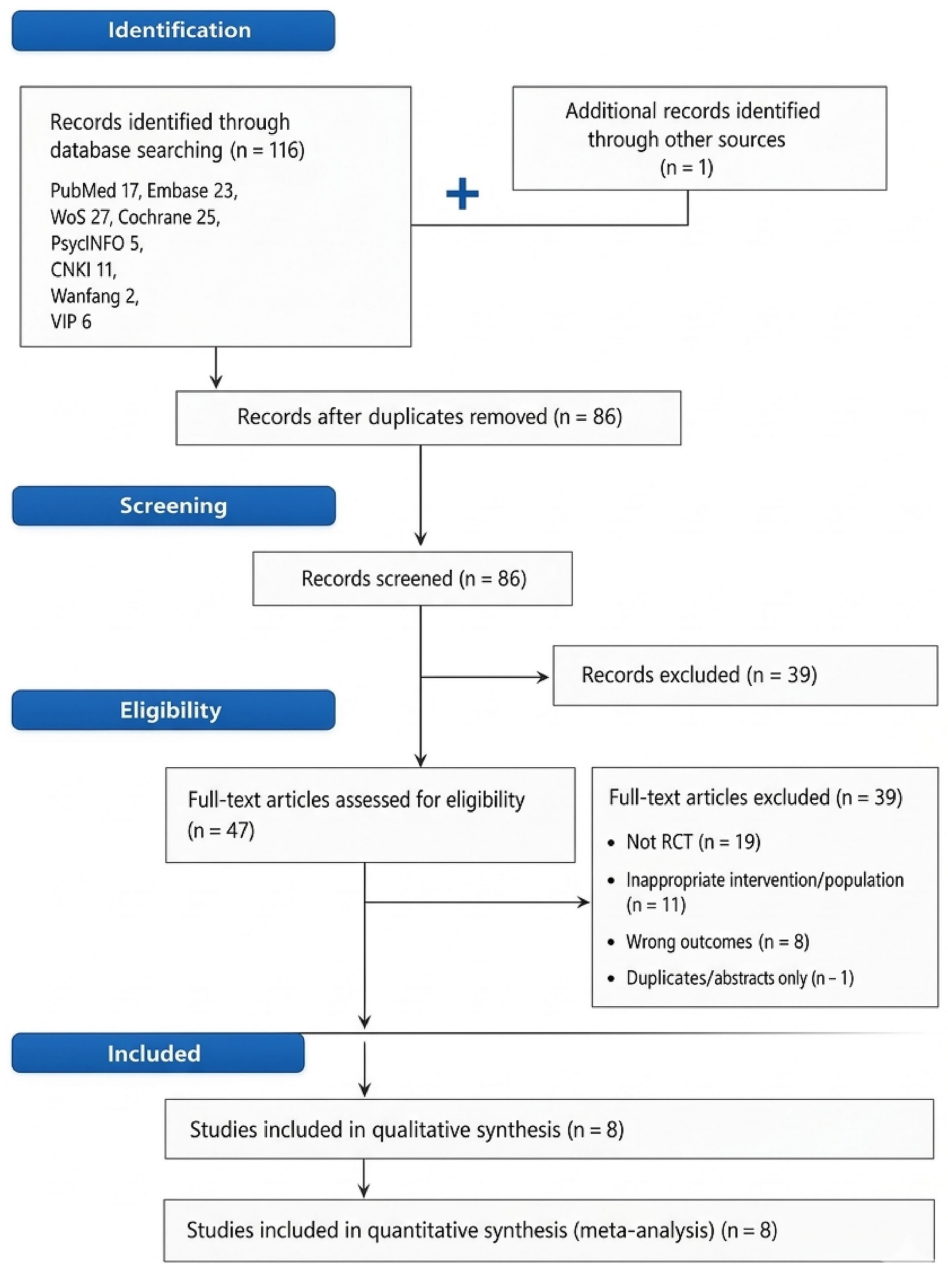
PRISMA2000 flow diagram.

A total of 117 records were initially identified through database searching (PubMed, Embase, Web of Science, Cochrane, PsycINFO, CNKI, WanFang, VIP) and manual search. After removing 31 duplicates, 86 records were screened based on titles and abstracts. Subsequently, 47 full-text articles were assessed for eligibility. Finally, 8 studies were included in this meta-analysis (3, 6, 11–16). The detailed selection process is presented in the PRISMA flow diagram (Figure 1).

### 3.2 Study characteristics

The characteristics of the 8 included studies are summarized in Table 1. The studies were published between 2019 and 2026, covering diverse geographic regions including Taiwan, Japan, Turkey, and Mainland China. A total of 585 participants were involved. Most studies focused on nursing home residents or community-dwelling older adults with depressive symptoms. The duration of horticultural therapy ranged from 6 to 20 weeks.

**Table 1:** Characteristics of the included studies in the meta-analysis.

Study	Country/region	Setting	Population	Outcome measure	Sample size (HT/CG)	Intervention type	Duration (weeks)	Frequency (sessions/week)	Session duration (min)	Baseline score (HT/CG)
Chu et al. (2019) (11)	Taiwan, China	Nursing home	Nursing home residents	GDS-15	75/75	Active gardening indoors	8	1	90–120	7.31 ± 0.37/5.44 ± 0.38
Makizako et al. (2019) (12)	Japan	Community	Community-dwelling older adults	GDS-15	26/28	Active Gardening outdoors	20	1	60–90	6.90 ± 4.70/6.40 ± 2.50
Hu (2020) (13)	Mainland China	Nursing home	Disabled older adults	GDS-30	32/32	Active gardening indoors	8	1	60	14.94 ± 9.05/17.47 ± 7.59
Yin (2021) (14)	Mainland China	Nursing home	Older adults in facilities	GDS-30	34/34	Active gardening indoors	6	1	30	4.79 ± 3.10/3.72 ± 6.79
Jiang et al. (2022) (15)	Mainland China	Nursing home	Older adults with dementia	GDS-30	30/30	Active gardening indoors and outdoors	8	1	90	17.40 ± 3.89/17.10 ± 4.03
Ren et al. (2022) (16)	Mainland China	Community	Rural empty-nesters	GDS-30	51/51	Active gardening indoors and outdoors	8	1	NA	11.27 ± 2.66/10.09 ± 2.50
Nishiwaki et al. (2025) (6)	Japan	Community	Community-dwelling older adults	GDS-15	18/23	Active gardening outdoors	20	1	60–90	6.60 ± 1.70/6,40 ± 2.40
Kabakci et al. (2026) (3)	Turkey	Nursing home	Nursing home residents	GDS-30	23/23	Active gardening outdoors	12	1	60	5.26 ± 2.89/5.28 ± 2.72

For Nishiwaki et al. (6), GDS-15 was measured as a secondary outcome. HT, Horticultural Therapy group; CG, Control Group; GDS, Geriatric Depression Scale (−15 for short form, −30 for long form); NA, Not Available (data not reported in the original study).

The baseline depressive symptoms varied across the included studies. According to the GDS scoring criteria, participants in most studies exhibited mild depressive symptoms. Notably, participants in the study by Yin (14) were within the normal range at baseline (Mean < 5), while Jiang et al. (15) included a population with more severe (moderate to severe) depressive symptoms. This baseline variation likely contributed to the observed heterogeneity in treatment effects.

### 3.3 Risk of Bias assessment

**Figure 2:**
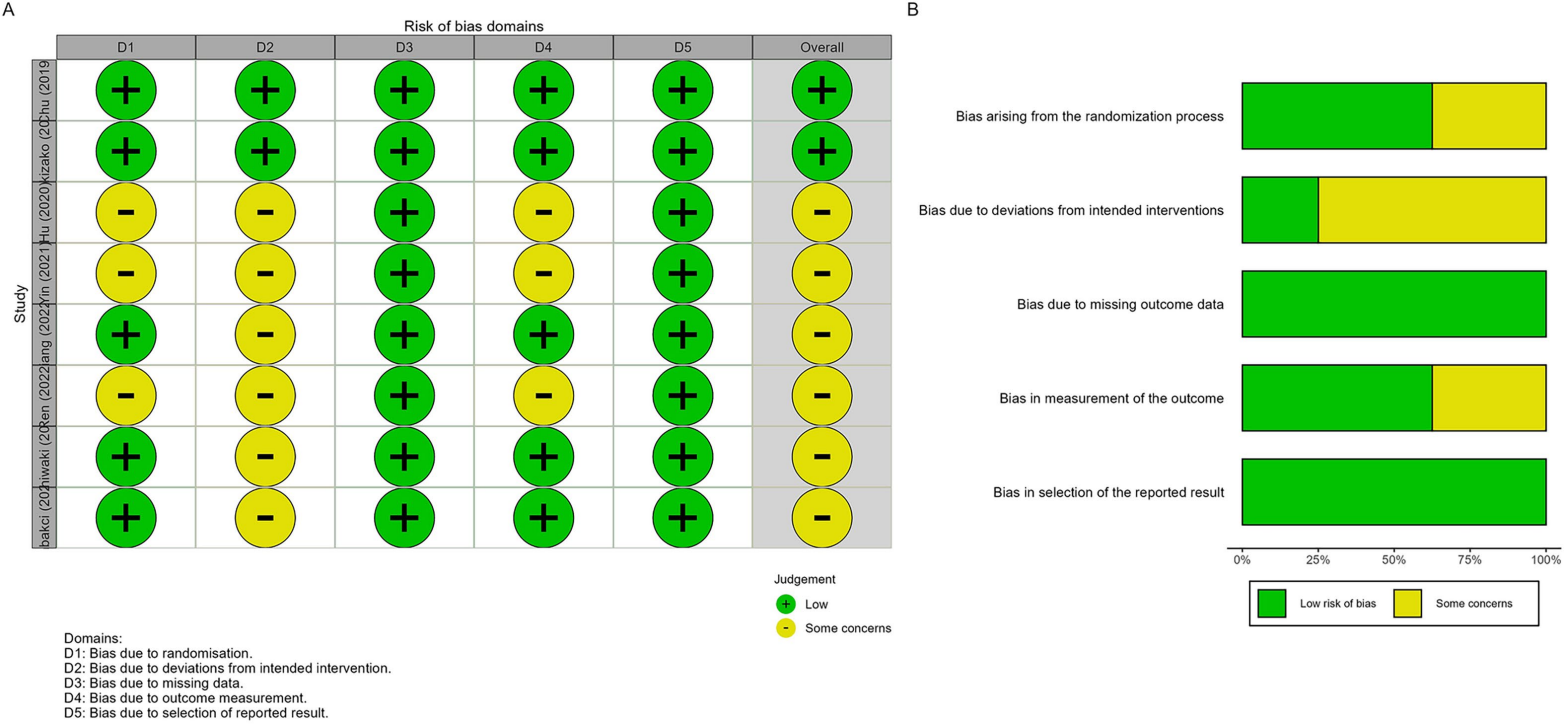
Risk of bias assessment of the included studies. **(A)** Traffic light plot presenting the risk of bias for each individual study across five domains. **(B)** Summary plot showing the overall percentage of studies at low risk, some concerns, and high risk of bias for each domain. Domains: D1, Randomization process; D2, Deviations from intended interventions; D3, Missing outcome data; D4, Measurement of the outcome; D5, Selection of the reported result.

The risk of bias assessment using the Cochrane RoB 2.0 tool is shown in Figure 2. Overall, 2 studies (11, 12) were rated as “low risk,” while the remaining 6 studies were rated as having “some concerns.” The primary source of bias was the lack of blinding of participants and personnel (Domain 2), which is common in social and behavioral interventions like horticultural therapy.

### 3.4 Primary outcome depression scores

**Figure 3:**
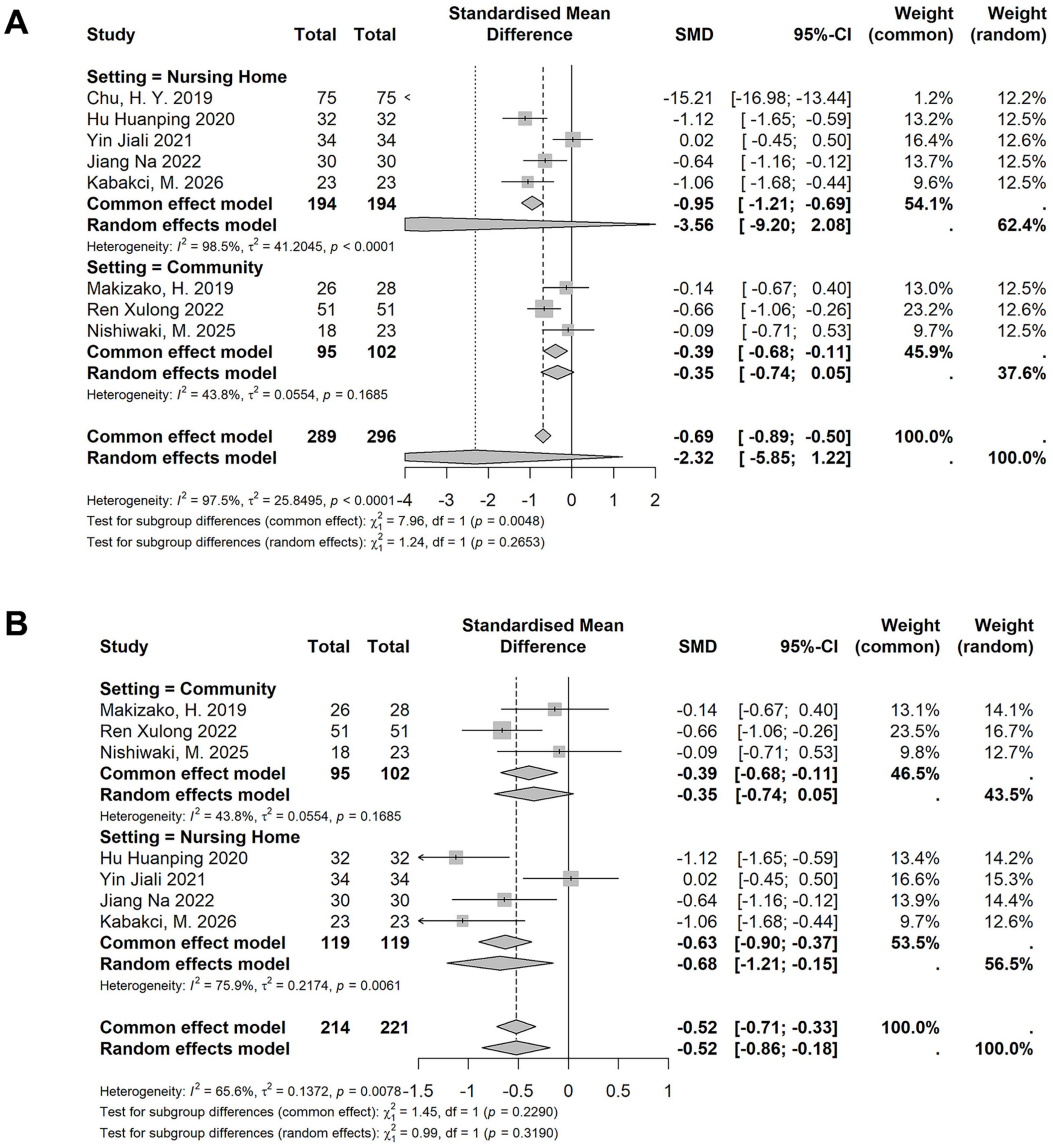
Forest plots of horticultural therapy efficacy: **(A)** primary analysis including all eight RCTs showing extreme heterogeneity; **(B)** sensitivity analysis after excluding the statistical outlier (11).

The initial meta-analysis of all 8 studies showed a significant reduction in depression scores following horticultural therapy. However, extreme heterogeneity was observed (I^2^ = 97.5%, *p* < 0.0001). Visual inspection of the forest plot (Figure 3A) identified one study (11) as a major outlier, reporting an unusually large effect size (SMD = −2.32).

### 3.5 Sensitivity analysis

Initial pooling of all eight studies revealed extreme heterogeneity (I^2^ = 97.5%,) and a non-significant overall effect (SMD = −2.32, 95% CI: −5.85 to 1.22), primarily driven by the extreme effect size reported in Chu et al. (11). The leave-one-out sensitivity analysis (presented in Figures 3A,B) demonstrated that Chu et al. (11) was a major outlier; its exclusion reduced from 97.5 to 65.6%. The antidepressant effect remained robust and statistically significant in the seven-study dataset (SMD = −0.52, *p* = 0.0078), confirming the stability of the core findings after addressing the outlier-driven variance.

### 3.6 Subgroup analysis

To further investigate the potential moderators of horticultural therapy’s effect on depression and to explore the sources of heterogeneity, several subgroup analyses were performed based on geographical location, assessment scales, total intervention dose, setting, and duration (Supplementary Table 2).

Regarding geographical regions, no significant difference was observed (*p* = 0.652) between studies conducted in China (SMD = −0.59, 95% CI: −1.05 to −0.14) and other countries (SMD = −0.42, 95% CI: −1.03 to 0.19), indicating that the antidepressant effect of horticultural therapy is consistent across different cultural contexts.

For the assessment scales, a marginal difference was found (*p* = 0.055). Studies utilizing the GDS-30 reported a more pronounced effect (SMD = −0.67, 95% CI: −1.07 to −0.27) compared to those using the GDS-15 (SMD = −0.12, 95% CI: −0.52 to 0.29). This interpretation warrants caution and should be framed as a marginally significant trend, indicating that the 30-item version of the GDS might have a potential inclination toward higher sensitivity in capturing psychological improvements.

In terms of total intervention dose, the results remained robust (*p* = 0.912). Low-dose interventions (≤ 600 min) yielded an SMD of −0.54 (95% CI, −1.67 to 0.58), which was comparable to high-dose interventions (> 600 min, SMD = −0.48, 95% CI: −0.91 to −0.04). This implies that relatively brief horticultural programs can be as effective as more intensive ones.

Additionally, no significant differences were found between settings (*p* = 0.201), although the effect size in nursing homes (SMD = −0.81) appeared larger than in community settings (SMD = −0.35). Finally, intervention duration did not significantly moderate the effect (*p* = 0.453), with both short-term (≤ 8 weeks, SMD = −0.66) and longer-term interventions (> 8 weeks, SMD = −0.40) showing significant reductions in depression scores.

### 3.7 Dose-response meta-analysis

To further explore the relationship between the total intervention dose and the antidepressant effect, a dose–response meta-analysis was conducted using restricted cubic splines with three knots. Six studies providing specific total duration data (ranging from 180 to 1,500 min) were included in this model.

**Figure 4:**
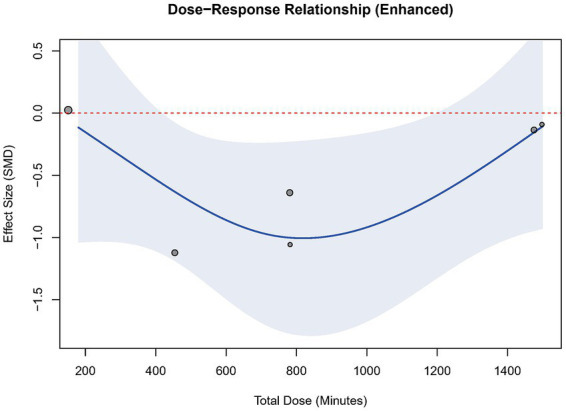
Dose–response relationship between total intervention dose and depression scores.

The analysis revealed a non-linear trend between the total dose of horticultural therapy and the reduction in depression scores, which approached statistical significance (p_non-linear_ = 0.076; Supplementary Table 3). While not meeting the conventional *p* < 0.05 threshold, the U-shaped curve provides a preliminary visualization of how HT intensity may relate to efficacy (Figure 4).

#### 3.7.1 Initial benefit

In the lower dose range (180 to 800 min), the standardized mean difference (SMD) decreased as the dose increased, indicating that greater time spent in horticultural activities yielded stronger antidepressant effects.

#### 3.7.2 Optimal threshold

The therapeutic benefit appeared to peak at approximately 700–800 min of total intervention (SMD reached its lowest point). Diminishing Returns: Beyond the 1,000-min mark, the curve showed an upward trend, suggesting that further increasing the intervention duration did not lead to additional symptom reduction and might even result in diminishing returns.

The ANOVA results for the spline model confirmed the marginal non-linearity (*F* = 7.09, *p* = 0.076; Supplementary Table 3), suggesting that the intensity of horticultural therapy and its clinical outcomes do not follow a simple linear progression.

### 3.8 Publication bias

Potential publication bias was evaluated using both visual and statistical methods. The funnel plot (Supplementary Figure 1) showed a generally symmetrical distribution of the individual studies around the pooled effect size. Statistical confirmation was provided by Egger’s linear regression test (t = −0.08, *p* = 0.936) and Begg’s rank correlation test (Z = −0.45, *p* = 0.652). Both tests yielded non-significant results (*p* > 0.05), indicating no evidence of significant publication bias in this meta-analysis. These findings suggest that the observed antidepressant effect of horticultural therapy is robust and unlikely to be driven by small-study effects or the selective publication of positive results.

## 4 Discussion

The current meta-analysis of randomized controlled trials (RCTs) confirms that horticultural therapy (HT) is an effective non-pharmacological intervention for reducing depressive symptoms in older adults. A key highlight of our study is the identification of a U-shaped dose–response relationship, with the potential exploratory antidepressant benefit occurring at a cumulative dose of approximately 700–800 min.

### 4.1 The “U-shaped” dose-response and the therapeutic window

The non-linear trend (p_non-linear_ = 0.076) observed in our spline model should be strictly interpreted as exploratory and hypothesis-generating, suggesting that the intensity of HT and its clinical outcomes do not follow a simple linear progression. In the initial stage (from 180 to 800 min), we observed that increasing the cumulative dose significantly enhanced the reduction of depression scores. This is consistent with previous evidence indicating that even light-intensity physical activity is associated with improved biological markers, such as lower C-reactive protein and reduced insulin resistance in older populations (17). Research has shown that achieving a threshold of at least 300 min per week of such activity can lead to more favorable health outcomes and a lower prevalence of chronic diseases (17). However, our curve shows a trend of “diminishing returns” or even a slight rebound after the 1,000-min mark. This phenomenon can be explained by the unique physiological and functional profiles of the geriatric population. While moderate-to-higher levels of activity are generally effective for maintaining functional independence, there exists a specific threshold beyond which the risks may outweigh the benefits (7). For frail older adults, excessive physical or cognitive demands from prolonged horticultural activities may exceed their physiological rationale, potentially leading to adverse events or fatigue that counteracts the psychological gains (8). Therefore, 700–800 min (approximately 60–90 min per week for 8–12 weeks) appears to be the “sweet spot” that maximizes therapeutic efficacy while respecting the physical limits of the older population. The marginal significance is likely attributable to the small sample of studies (*n* = 6) providing precise dosage data, which may lack sufficient statistical power to reach conventional significance.

### 4.2 Sensory stimulation and scale sensitivity: the multi-dimensional impact

A notable finding in our subgroup analysis was the discrepancy between the GDS-30 (SMD = −0.67) and GDS-15 (SMD = −0.12) (*p* = 0.055). This suggests that HT provides a holistic psychological benefit that is more effectively captured by longer assessment tools. Unlike traditional physical activities, HT is a comprehensive intervention involving multi-sensory stimulation (14, 15). From the perspective of the Biophilia Hypothesis, humans possess an innate tendency to seek connections with nature, which can trigger positive emotional responses (4). According to Stress Reduction Theory (SRT), exposure to natural elements like plants can rapidly lower physiological arousal and reduce cortisol levels, thereby alleviating the physical symptoms of depression (5). The “five-senses” approach, as detailed by Yin (14), leverages these mechanisms by engaging vision, olfaction, and touch. The GDS-30, which includes more items related to social withdrawal, cognitive hopelessness, and loss of vigor, may be more sensitive to these nuanced improvements in self-worth and environmental engagement facilitated by Attention Restoration Theory (ART) (18, 19).

### 4.3 Subgroup differences: institutional context and cultural resilience

The efficacy of HT remained robust across different settings, but was particularly pronounced in institutionalized older adults. For those living in nursing homes, social isolation and “environmental boredom” are primary risk factors for depression (20). HT functions not only as a physical activity but also as a “social lubricant,” fostering peer interaction and collective identity (21). Lu et al. demonstrated that HT significantly increases social engagement and positive effects in residents with dementia (22), a finding that aligns with our subgroup results in nursing home settings.

Geographically, the strong positive outcomes observed in Asian studies (e.g., China, Japan, Turkey) might reflect a cultural resonance. In many Asian philosophies, the concept of “nature-man harmony” provides a fertile psychological ground for HT to succeed (23). Furthermore, systematic reviews of HT in East Asia have consistently shown its superiority in improving geriatric mental health compared to waiting-list controls (24). However, the relative scarcity of European RCTs in our sample underscores the need for more cross-cultural validation to confirm if these benefits are universally applicable regardless of cultural perceptions of nature.

### 4.4 Heterogeneity and methodological robustness

Metas of complex interventions like HT often encounter significant heterogeneity (25). In our study, the initial extreme heterogeneity (I^2^ = 97.5%) was largely driven by Chu et al. (11). According to Higgins et al. (9), identifying and managing such outliers is crucial for ensuring the integrity of the pooled estimate. The exclusion of Chu et al. (11) was necessitated by both its extreme statistical deviation (standardized residual > ± 3) and clinical implausibility. Specifically, the unusual deterioration of the control group in Chu et al.—where GDS-15 scores worsened from 5.44 to 8.08 within 8 weeks—mathematically inflated the effect size to an implausible SMD of −15.21. By documenting the full process from initial inclusion to systematic diagnostic exclusion, we ensured that our final synthesis is grounded in clinically representative data rather than statistical anomalies.

Despite the exclusion of the primary outlier, a moderate-to-high residual heterogeneity (I^2^ = 65.6%) persisted among the remaining seven studies, reflecting the inherent clinical diversity of HT. While the intervention protocols across all included trials consistently focused on active gardening tasks—involving physical engagement and goal-oriented activities—the specific methodologies (e.g., choice of plant species, specific tools, and social grouping) varied across studies. Due to manuscript length limitations, an exhaustive list of these granular protocols is not feasible; however, these methodological nuances undoubtedly contribute to the observed variance.

The inclusion of cognitively impaired populations alongside cognitively intact peers introduces further complexity, a point of concern regarding study comparability. For participants with dementia, active HT likely serves as a vital non-pharmacological neurosensory stimulation, targeting cognitive maintenance and behavioral stabilization. Conversely, for community-dwelling seniors or rural “empty-nesters,” the therapeutic benefit may stem more from social reintegration and the alleviation of loneliness. Furthermore, the variation in baseline depression severity—ranging from normal mood states in Yin (14) to moderate-to-severe depression in Jiang et al. (15)—may lead to a “floor effect,” where the inclusion of subclinical populations dilutes the estimated treatment effect.

In summary, while these clinical and population-based differences contribute to the statistical heterogeneity, they also highlight the versatility of active HT across the geriatric mental health spectrum. By utilizing a random-effects model to account for these diverse cognitive and social contexts, our pooled SMD of −0.52 provides a robust and realistic estimate of the typical benefit of HT in diverse clinical settings.

### 4.5 Limitations

Despite the rigorous methodology and the novel identification of a non-linear dose–response relationship, several limitations should be acknowledged.

First, the total number of included RCTs was relatively small (*n* = 7 after outlier removal), which may limit the statistical power to detect more subtle moderators or a highly significant non-linear-value. While the trend for a U-shaped relationship was clear (*p* = 0.076), a larger pool of studies would strengthen the precision of the identified optimal dose (26).

Second, there was significant clinical and methodological diversity in the horticultural therapy (HT) protocols. Although we addressed this through random-effects modeling and subgroup analysis, variations in plant types, outdoor versus indoor settings, and active gardening versus passive sensory stimulation may still contribute to the residual heterogeneity (27).

Third, the geographic distribution of the included studies was predominantly centered in Asian countries (e.g., China, Japan, Turkey). Cultural perceptions of nature and gardening can vary significantly, and the findings may not be fully generalizable to Western geriatric populations (23).

Finally, most studies assessed outcomes immediately after the intervention period (ranging from 6 to 20 weeks). Therefore, the long-term sustainability of the antidepressant effects of HT remains uncertain and warrants further longitudinal investigation.

## 5 Conclusion

In conclusion, our systematic review and dose–response meta-analysis provide robust evidence that horticultural therapy is an effective non-pharmacological intervention for alleviating depression in older adults. The most critical exploratory finding is the non-linear relationship between the intervention dose and antidepressant efficacy, identifying a potential therapeutic window of approximately 700–800 min associated with favorable clinical outcomes. Given the nature of the available evidence, this dose–response trend should be considered exploratory and hypothesis-generating, warranting further validation in larger clinical cohorts.

To optimize potential therapeutic benefits, clinical practitioners and older population care facility managers could consider integrating this preliminary cumulative dose window into their program designs while remaining vigilant of potential physical fatigue in frail individuals, which could lead to diminishing returns. Future research should focus on high-quality, multi-center RCTs with longer follow-up periods and standardized protocols to further refine and validate the dose–response framework for horticultural therapy in diverse cultural contexts.

## Data Availability

Publicly available datasets were analyzed in this study. This data can be found at: the primary data analyzed in this study are derived from peer-reviewed articles publicly available in the databases PubMed, Embase, Web of Science, Cochrane Library, PsycINFO, CNKI, WanFang, and VIP. All standardized data extracted from these studies and the results of the restricted cubic spline (RCS) analysis are included within this article and its Supplementary material. No new datasets were generated or deposited in external repositories.

## Supplementary material

SUPPLEMENTARY FIGURE 1 Funnel plot.
