# Supplementary figures and images for: Dose-response efficacy of horticultural therapy for geriatric depression: a systematic review and meta-analysis of randomized controlled trials
Source: Front Public Health. 2026 Jul 17;14:1824111. doi: 10.3389/fpubh.2026.1824111 (PMC13423710; doi:10.3389/fpubh.2026.1824111)

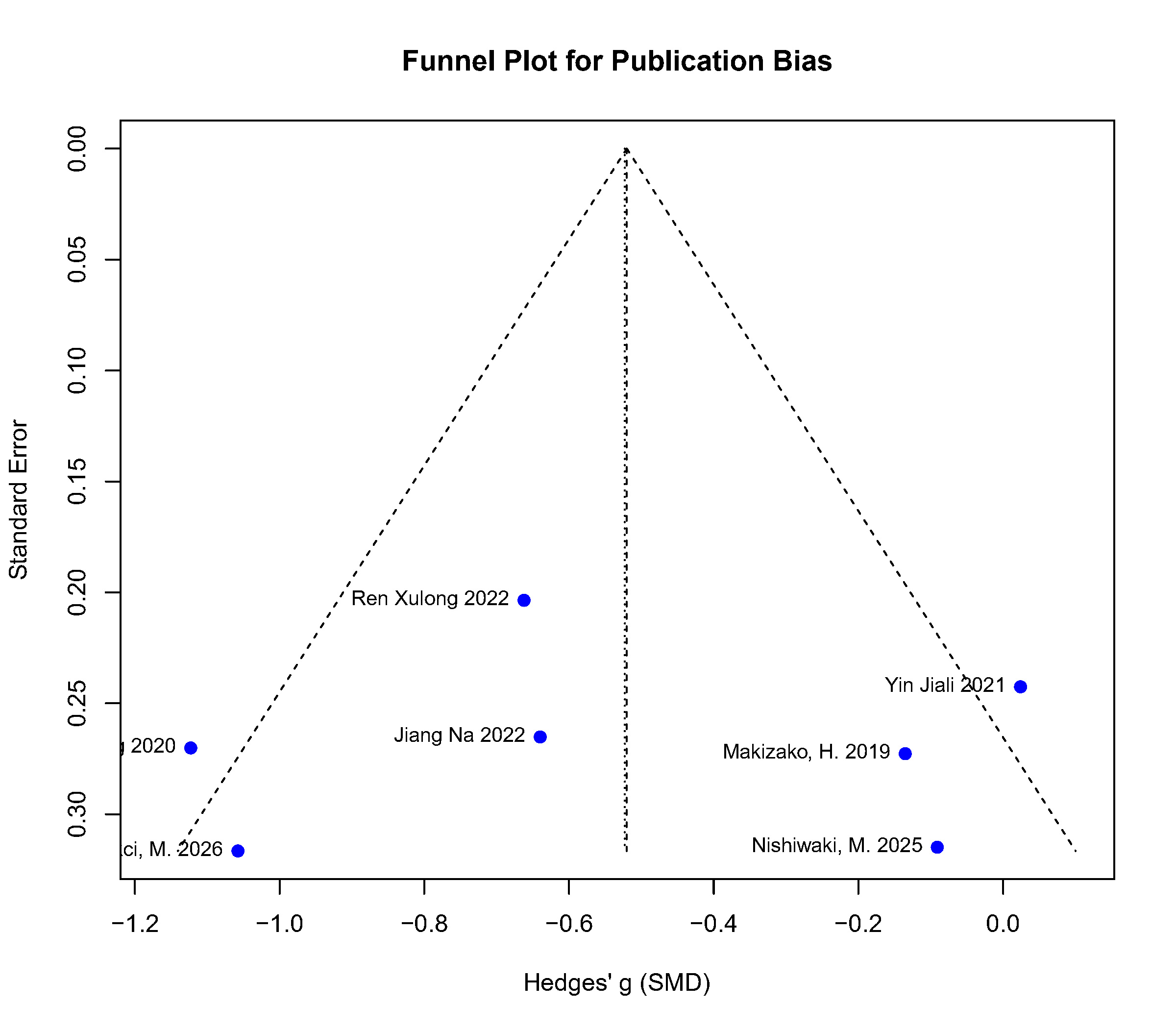

Supplement: SUPPLEMENTARY FIGURE 1 — Funnel plot. [file Image_1.JPEG]
