# Supplementary material for: Dose-response efficacy of horticultural therapy for geriatric depression: a systematic review and meta-analysis of randomized controlled trials
Source: Front Public Health. 2026 Jul 17;14:1824111. doi: 10.3389/fpubh.2026.1824111 (PMC13423710; doi:10.3389/fpubh.2026.1824111)
Supplement: Supplementary file 2 [file Table_1.DOCX]

**Supplementary Table1 Detailed Search Strategies**

| **Item** | **Database** | **Records Identified** | **Format** | **Remarks** | **Search String** |
| --- | --- | --- | --- | --- | --- |
| 1 | PubMed | 17 | .txt |  | (((("Horticultural Therapy"[MeSH Terms]) OR ("Gardening"[MeSH Terms])) OR (Horticultural Therapy[Title/Abstract] OR Gardening[Title/Abstract] OR Garden-based[Title/Abstract] OR Nature-based therapy[Title/Abstract] OR Therapeutic landscapes[Title/Abstract])) AND ((("Depression"[MeSH Terms]) OR ("Depressive Disorder"[MeSH Terms])) OR (Depression[Title/Abstract] OR Depressive Symptoms[Title/Abstract] OR Emotional Depression[Title/Abstract])) AND ((("Aged"[MeSH Terms]) OR ("Older Adults"[Title/Abstract])) OR (Elderly[Title/Abstract] OR Geriatric[Title/Abstract] OR Seniors[Title/Abstract]))) AND ((randomized controlled trial[Publication Type]) OR (randomized[Title/Abstract] OR placebo[Title/Abstract] OR trial[Title/Abstract])) |
| 2 | EMBASE | 23 | .ris |  | ('horticultural therapy'/exp OR 'gardening'/exp OR 'horticultural therapy':ti,ab OR 'gardening':ti,ab OR 'nature-based intervention':ti,ab) AND ('depression'/exp OR 'depressive disorder'/exp OR 'depression':ti,ab OR 'depressive symptoms':ti,ab) AND ('aged'/exp OR 'elderly':ti,ab OR 'older adult':ti,ab OR 'senior citizen':ti,ab) AND ('randomized controlled trial'/exp OR 'randomization':ti,ab OR 'randomised':ti,ab) |
| 3 | Web of Science | 27 | .txt |  | ("Horticultural Therap*" OR "Garden*" OR "Nature-based intervention*") AND ("Depression" OR "Depressive Symptom*" OR "Depressive Disorder*") AND ("Older adult*" OR "Elderly" OR "Aged" OR "Geriatric*") AND ("Randomized controlled trial*" OR "Randomised" OR "Clinical trial*") |
| 4 | Cochrane Library | 25 | .ris |  | (MeSH descriptor: [Horticultural Therapy] explode all trees OR MeSH descriptor: [Gardening] explode all trees OR "horticultural therapy":ti,ab,kw OR "gardening":ti,ab,kw) AND (MeSH descriptor: [Depression] explode all trees OR MeSH descriptor: [Depressive Disorder] explode all trees OR "depression":ti,ab,kw OR "depressive symptoms":ti,ab,kw) AND (MeSH descriptor: [Aged] explode all trees OR "older adults":ti,ab,kw OR "elderly":ti,ab,kw) |
| 5 | PsycINFO | 5 | .ris |  | ("Horticultural Therapy" OR "Gardening") AND ("Depression" OR "Depressive Symptoms") AND ("Older adults" OR "Elderly" OR "Aged") AND ("Randomized Clinical Trials" OR "Randomized") |
| 6 | CNKI | 11 | .enw |  | 主题=(园艺治疗 + 园艺疗法 + 园艺活动) * (抑郁 + 忧郁 + 心理健康) * (老年人 + 老人) * (随机 + 对照 + 试验) |
| 7 | WanFang | 2 | .txt |  | 题名或关键词:("园艺治疗" OR "园艺疗法" OR "园艺活动")) AND (题名或关键词:("抑郁" OR "忧郁")) AND (题名或关键词:("老年人" OR "老人")) AND (题名或关键词:("随机" OR "对照") |
| 8 | VIP | 6 | .txt |  | (M=(园艺治疗 OR 园艺疗法 OR 园艺活动)) AND (M=(抑郁 OR 忧郁)) AND (M=(老年人 OR 老人)) AND (M=(随机 OR 对照)) |
| 9 | Manual Search | 1 |  |  |  |
| **A** | Total Records Identified | 117 |  |  |  |
| **B** | Duplicates Removed | 31 |  | Via Endnote |  |
| **C** | Unique Records for Screening | 86 |  | Target for Title/Abstract screening |  |
