# Supplementary material for: Dose-response efficacy of horticultural therapy for geriatric depression: a systematic review and meta-analysis of randomized controlled trials
Source: Front Public Health. 2026 Jul 17;14:1824111. doi: 10.3389/fpubh.2026.1824111 (PMC13423710; doi:10.3389/fpubh.2026.1824111)
Supplement: Supplementary file 3 [file Table_2.DOCX]

**Supplementary Table 2**

**Subgroup analysis of horticultural therapy on depression in older adults**

| **Moderator** | **No. of studies (k)** | **Effect size (g)** | **95% CI** | **P_subgroup_​** | **I^2^ (%)** |
| --- | --- | --- | --- | --- | --- |
| **Country** |  |  |  | 0.652 |  |
| China | 4 | -0.59 | [-1.05, -0.14] |  | 71.30% |
| Other countries | 3 | -0.42 | [-1.03, 0.19] |  | 67.90% |
| **Assessment Scale** |  |  |  | 0.055 |  |
| GDS-30 | 5 | -0.67 | [-1.07, -0.27] |  | 67.90% |
| GDS-15 | 2 | -0.12 | [-0.52, 0.29] |  | 0.00% |
| **Total Dose** |  |  |  | 0.912 |  |
| ≤ 600 min | 2 | -0.54 | [-1.67, 0.58] |  | 90.00% |
| > 600 min | 4 | -0.48 | [-0.91, -0.04] |  | 56.00% |
| **Setting** |  |  |  | 0.201 |  |
| Nursing Home | 4 | -0.81 | [-1.41, -0.21] |  | 75.20% |
| Community | 3 | -0.35 | [-0.74, 0.05] |  | 43.80% |
| **Duration** |  |  |  | 0.453 |  |
| ≤ 8 weeks | 4 | -0.66 | [-1.06, -0.26] |  | 55.40% |
| > 8 weeks | 3 | -0.4 | [-0.74, -0.06] |  | 67.90% |
