# Supplementary material for: Dose-response efficacy of horticultural therapy for geriatric depression: a systematic review and meta-analysis of randomized controlled trials
Source: Front Public Health. 2026 Jul 17;14:1824111. doi: 10.3389/fpubh.2026.1824111 (PMC13423710; doi:10.3389/fpubh.2026.1824111)
Supplement: Supplementary file 4 [file Table_3.DOCX]

**Table 3. Polynomial Regression Analysis of the Dose-Response Relationship**

| Variable | Coefficient (β) | Standard Error (SE) | t-value | P-value |
| --- | --- | --- | --- | --- |
| Total Dose (Linear) | -0.0031 | 0.0012 | -2.58 | 0.081 |
| Total Dose (Non-linear) | 0.0058 | 0.0022 | 2.66 | 0.076 |
| Model Overall | -- | -- | F=3.82 | 0.149 |
